# Life Cycle Greenhouse Gas Emissions of Brazilian Sugar Cane Ethanol Evaluated with the GREET Model Using Data Submitted to RenovaBio

**DOI:** 10.1021/acs.est.2c08488

**Published:** 2023-08-01

**Authors:** Xinyu Liu, Hoyoung Kwon, Michael Wang, Don O’Connor

**Affiliations:** † Systems Assessment Center, Energy Systems and Infrastructure Analysis Division, Argonne National Laboratory, 9700 S Cass Avenue, Lemont, Illinois 60439, United States; ‡ Sustainability Sciences Division, Indigo Ag, Inc., 500 Rutherford Avenue, Boston, Massachusetts 02129, United States; § S&T Squared Consultants Inc., 11657 Summit Crescent, Delta, BC V4E2Z2, Canada

**Keywords:** Brazilian sugar cane ethanol, RenovaBio, life cycle analysis, land use change, greenhouse gas emissions

## Abstract

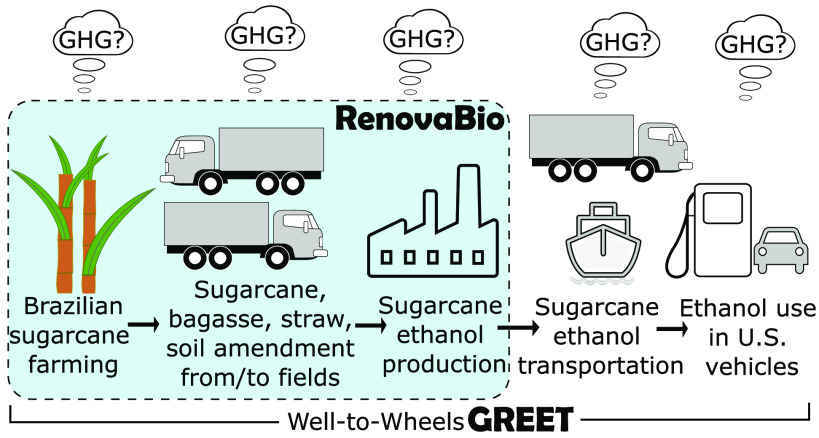

Brazil is the second-largest ethanol producer in the world, primarily using sugar cane as feedstock. To foster biofuel production, the Brazilian government implemented a national biofuel policy, known as RenovaBio, in which greenhouse gas (GHG) emission reduction credits are provided to biofuel producers based on the carbon intensities (CI) of the fuels they produce. In this study, we configured the GREET model to evaluate life cycle GHG emissions of Brazilian sugar cane ethanol, using data from 67 individual sugar cane mills submitted to RenovaBio in 2019/2020. The average CI per megajoule of sugar cane ethanol produced in Brazil for use in the U.S. was estimated to be 35.2 g of CO_2_ equivalent, a 62% reduction from U.S. petroleum gasoline blendstock without considering the impacts of land use change. The three major GHG sources were on-field N_2_O emissions (24.3%), sugar cane farming energy use (24.2%), and sugar cane ethanol transport (19.3%). With the probability density functions for key input parameters derived from individual mill data, we performed stochastic simulations with the GREET model to estimate the variations in sugar cane ethanol CI and confirmed that despite the larger variations in sugar cane ethanol CI, the fuel provided a robust GHG reduction benefit compared to gasoline blendstock.

## Abstract 2

Life cycle analysis using data from 67 individual plants collected through Brazil’s National Biofuel Program, RenovaBio, demonstrates greenhouse gas mitigation potential from Brazilian sugar cane ethanol production for use in the United States.

## 1 Introduction

Brazil is the second-largest producer of ethanol after the United States (U.S.), with a production output of 7.93 billion gallons in 2020 and representing 30% of worldwide ethanol production output.^1^ The Brazilian ethanol industry primarily uses sugar cane as feedstock, while its U.S. counterpart mainly uses corn. To foster biofuel production, the Brazilian government introduced RenovaBio, a national biofuel policy.^2^ Under RenovaBio, greenhouse gas (GHG) reduction credits, also known as CBios, are calculated for individual biofuel producers on a life cycle basis. Biofuel producers need to report life cycle inventory (LCI) data that are verified by a third-party inspection firm. Annual decarbonization targets are established for fuel distributors, who are obliged to purchase CBios either directly from biofuel producers or indirectly from the secondary stock market, according to guidelines established by the National Agency for Petroleum, Natural Gas and Biofuels through Resolution 758/2018. In 2019/2020, data representing a total of 153 million metric tons of sugar cane, or 24% of the sugar cane produced in Brazil in 2019, were collected from 67 mills.^3^ Under RenovaBio, the GHG reduction credit of biofuels needs to be quantified by life cycle analysis (LCA) methodology, accounting for the material/energy inputs and the associated GHG emissions along the ethanol supply chain.

Extensive studies have been published to quantify the GHG reduction potential of Brazilian sugar cane ethanol. Pereira et al.^4^ and Khatiwada et al.^5^ compared the GHG emissions results of Brazilian sugar cane ethanol from three widely used LCA models employed by different regulatory agencies, namely the Greenhouse Gases, Regulated Emissions, and Energy Use in Technologies (GREET) model for the U.S.,^6^ BioGrace (www.biograce.net) for the European Union (EU), and GHGenius^7^ for Canada. The calculated sugar cane ethanol GHG emissions or carbon intensities (CI) in the Pereira et al. study ranged from 16 to 45 g of CO_2_ equivalent (g of CO_2_e) per megajoule (MJ), based on a lower heating value (LHV) of 80.5 MJ/gallon of ethanol.^4^ The large variations in sugar cane ethanol CI are due to different assumptions made by these models regarding (1) sugar cane farming parameters (i.e., agricultural practices, fertilizer and energy use, percentage of open-field straw burning, and N_2_O emissions from fertilizers/residues), (2) sugar cane mill operation parameters (i.e., sugar cane transportation distance, products profile, fossil/renewable energy consumption, and enzyme and chemical use), (3) transportation modes and distances for ethanol shipping from Brazil to destination countries, (4) allocation methods for co-products (i.e., energy allocation, economic value allocation, and system expansion), (5) uncertainties associated with the economic modeling approach for induced land use change (LUC), and (6) different global warming potentials (GWP) of GHGs employed in different impact assessment methods.^8^ After the harmonization of assumptions using parameters from Virtual Sugar Cane Biorefinery (VSB), a Brazilian platform for sugar cane ethanol assessments, Pereira et al. reported a significant reduction in the sugar cane CI range (16–17 g of CO_2_e/MJ).^4^

Despite different assumptions made, studies of Brazilian sugar cane ethanol production reported deep well-to-wheels (WTW) GHG emissions reductions compared to conventional gasoline (excluding LUC emissions): 75% by Macedo et al.,^9^ 78% by Wang et al.,^10^ 81% by Luo et al.,^11^ 74% by Crago et al.,^12^ 66–71% by Wang et al.,^13^ 60–90% by Chum et al.,^14^ 69–75% by Wang et al.,^15^ and 75% by Klein et al.^16^ Including the LUC emissions, the GHG reduction potential of sugar cane ethanol decreased. Wang et al. used an LUC emission of 16 g of CO_2_e/MJ for sugar cane ethanol, and the GHG reduction potential decreased to 40–62%.^13^ The U.S. Environmental Protection Agency (EPA) estimated the LUC emissions associated with Brazilian sugar cane ethanol production to be 4.5 g of CO_2_e/MJ. After including such emissions, the WTW GHG emissions reduction of Brazilian sugar cane ethanol is 61% compared to gasoline.^17^

Most of these studies used literature data for the input parameters. Seabra et al. conducted a sugar cane ethanol LCA using the latest data available from the Sugar Cane Research Center at the time, collected from sugar cane mills.^18^ They derived probability density functions (PDFs) for key sugar cane ethanol parameters based on collected data available from the industry. However, in the data set they used, mills were not required to disclose all information. For example, only 27 out of the 168 mills reporting sugar cane yield reported total diesel consumption during farming. Therefore, the collected data might not be representative. Moreover, the data were based on the 2008/2009 growing season, which does not reflect current practices. Furthermore, their study did not account for the use of biodiesel, which is currently mixed with fossil diesel at various blending ratios in the Brazilian market.

It is also worth mentioning that there was a significant mechanization uptrend for sugar cane collection during the past decade. The mechanization reduces sugar cane burning practices but increases diesel consumption. This may affect the WTW GHG emissions of ethanol produced.

Our current study advances the understanding of the sugar cane ethanol LCA by (1) compiling the recent LCI data for sugar cane farming and ethanol production, collected through RenovaBio for 67 sugar cane mills in 2019/2020; (2) deriving more representative PDFs for key LCA input parameters, since individual plants are required to disclose all necessary information to RenovaBio; and (3) performing an up-to-date literature review on the LUC emissions related to sugar cane ethanol production.

## 2 Materials and Methods

We configured and utilized the GREET model developed at Argonne National Laboratory to perform the LCA for Brazilian sugar cane ethanol, to quantify its GHG emissions reduction potential, and to identify emissions hot spots. Our focus is on GHG emissions, including CO_2_, CH_4_, and N_2_O, weighted by using their 100-year GWP, estimated in the Intergovernmental Panel on Climate Change (IPCC) Fifth Assessment Report (1, 30, and 265, respectively), to convert them to CO_2_e.^19,24^ For biogenic CH_4_ emissions from biomass combustion or burning, we accounted for its CO_2_ equivalent emissions while considering carbon uptake during biomass growth as credit.

**Figure 1:**
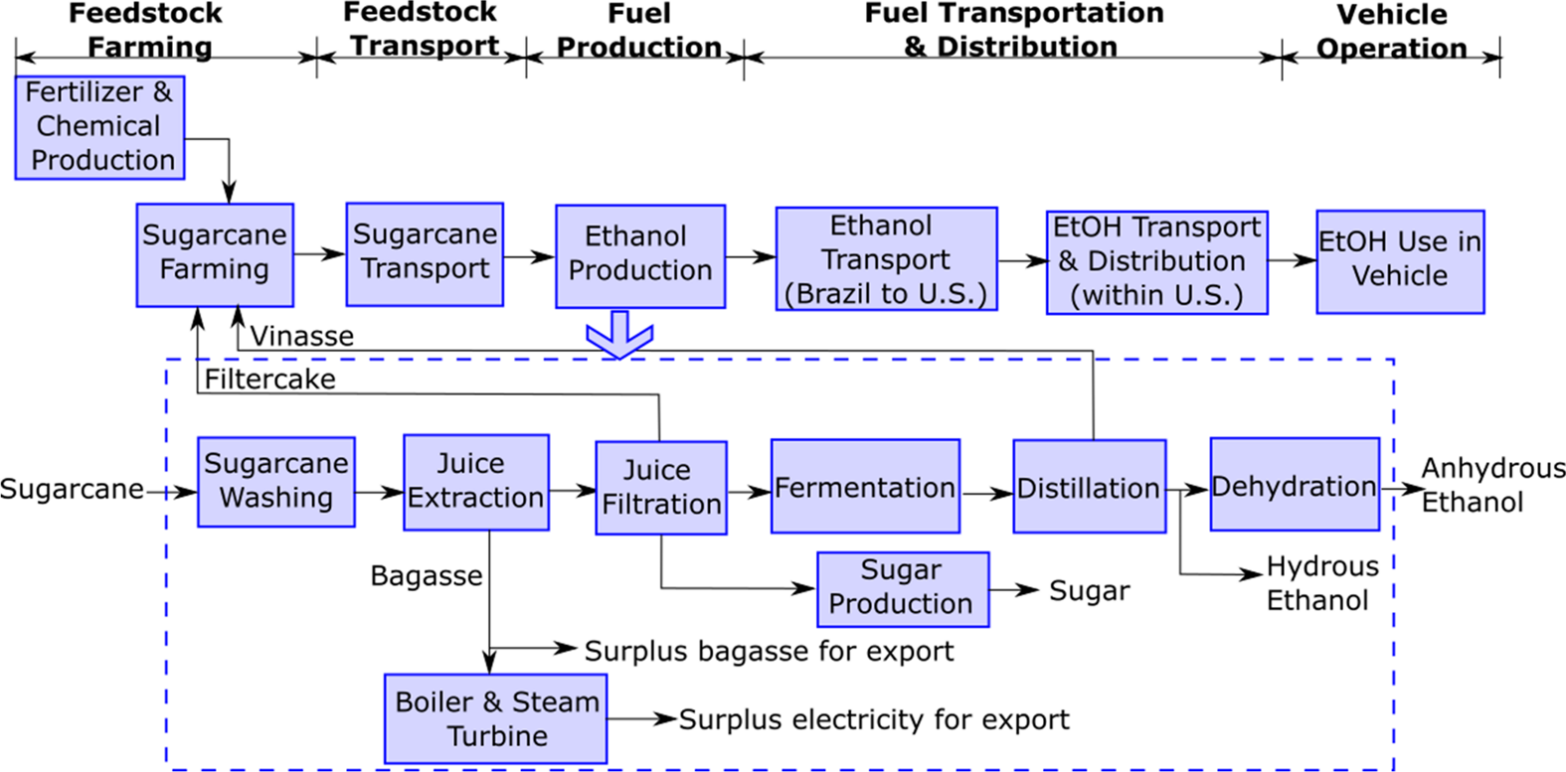
Schematic representation of the sugar cane ethanol (EtOH) pathway assessed by this study.

Our goal is to evaluate the WTW GHG emissions associated with sugar cane ethanol production in Brazil for use in the United States. Our system boundary covers chemical/fertilizer production in Brazil, farming, sugar cane transportation from fields to ethanol mills, ethanol production in Brazil, ethanol transportation from Brazil to U.S. ports and then to U.S. refueling stations, and the use of ethanol in U.S. vehicles, as shown in Figure 1. It is worth noting that this work comprises production data mostly on ethanol derived from the fermentation of sugar in sugar cane juice.

The WTW GHG emissions are calculated for a megajoule of anhydrous, undenaturated sugar cane ethanol (i.e., 0% of gasoline blendstock by volume added to ethanol as a denaturant) produced in Brazil and used in the U.S. The results are compared to those from U.S. gasoline blendstock on a per-megajoule energy basis. For the gasoline blendstock pathway, we adopted the WTW GHG emissions value from the GREET 2021 model without modifications.^6^

Sugar cane mills produce several other products, including sugars, anhydrous ethanol, hydrous ethanol, and electricity. The details of the co-product handling method and allocation procedure will be discussed in section 2.2.

The energy use and emissions associated with the manufacturing of farming equipment and the construction of sugar cane mills are excluded from this analysis due to their small contributions (∼2%) to the overall sugar cane ethanol CI.^9^

Since most activities along the sugar cane ethanol pathway occur in Brazil, we assumed that the electricity consumed is from the Brazilian grid, which relies heavily on hydroelectric power that has lower life cycle GHG emissions compared to the U.S. electricity grid (Table S1). The transmission and distribution loss for the Brazilian grid is 17%.^20^

Eight of the 67 sugar cane mills in our database chose to use the default application rates for synthetic fertilizers, organic fertilizers, and soil amendments provided by RenovaBio. We excluded those mills when calculating the production-weighted average application rates and shares and when deriving PDFs for key input parameters, since applying those conservative default rates would not represent actual practices and would most likely lead to penalized, high ethanol CI values.

### 2.1 Sugar Cane Farming

#### 2.1.1 Agrochemicals Manufacturing

The sugar cane mills reported synthetic nitrogen (N), phosphorus (P_2_O_5_), and potash (K_2_O) fertilizer use during sugar cane farming. Seven types of synthetic N fertilizers and four types of P_2_O_5_ fertilizers were used (Table S2). It is worth noting that monoammonium phosphate (MAP) and diammonium phosphate (DAP) serve as both nitrogen and phosphorus sources. To avoid double counting, we allocated 31.1% of the GHG emissions associated with MAP production to its use as N fertilizers, while the rest is allocated to its use as phosphorus fertilizers, based on the mass percents of N and P in MAP. For DAP, the allocation factor is 47.5% for N fertilizers.^21^ The manufacturing energy consumption data for chemicals/fertilizers were adopted from the GREET model.^22^

There are two major types of organic N fertilizers, namely filtercake and vinasse.^18^ Filtercake is the residue from sugar cane juice filtration, while vinasse is the remaining material from the distillation column bottoms after the desired product, ethanol, is removed. Their application rates and nitrogen contents are reported by the sugar cane mills. Since both are byproducts from sugar cane mills, we did not account for the GHG emissions associated with their production. However, we considered the GHG emissions due to their transportation from mills to the field, namely the open-channel transportation of vinasse and the truck transportation of filtercake.

Sugar cane farms reported the use of two types of limestone (dolomite and calcite) and gypsum as soil amendments. Since GREET 2021 did not differentiate between these limestone types, we assumed that both limestone types have the same LCI.^18,22^ We obtained the LCI data for gypsum production from GHGenius.^7^

The sugar cane mills have not reported their pesticide application rates. Therefore, we adopted the GREET default pesticide application rates for sugar cane farming.^6^

#### 2.1.2 On-Field Soil Emissions: N_2_O and CO_2_

N_2_O emissions from soils related to farming are empirically calculated with the IPCC Tier 1 methodology by using the amount of N inputs to soil and the conversion rates between different types of N sources to N_2_O. In this study, the covered sugar cane mills have not reported the straw yield in their fields. Therefore, we adopted a straw yield of 140 kg per metric ton of sugar cane.^13,18^ The amount of sugar cane straw collected from the fields is reported by the sugar cane mills, and we assumed that the rest was left in the fields, with their N content returned to soil. While improving soil quality, the N in sugar cane straw biomass would incur additional on-field N_2_O emissions. In addition, the N_2_O emissions from sugar cane root biomass are also accounted for, the N content of which is adopted from the RenovaBio calculator (version 7).^23^ Furthermore, N_2_O emissions are associated with the application of synthetic and organic N fertilizers.

In this analysis, we adopted the direct N_2_O emission factor (EF) of 1% from the 2019 IPCC report. On-field N_2_O emissions stem from the nitrification and denitrification of N in sugar cane biomass, including straw and root, and N in N fertilizers, including synthetic and organic. For indirect N_2_O emissions, we also relied on the IPCC estimate. IPCC estimated the volatilization rate for soil N to be 11%, with a volatilized N to N_2_O–N conversion rate of 1%. The leaching/runoff rate of soil N is estimated to be 24%, with a leached/runoff N to N_2_O–N conversion rate of 1.1%. Thus, the indirect N_2_O EF is estimated to be 0.374% (11% × 1% + 24% × 1.1%) for synthetic N fertilizer. Since there are no volatilization-related N_2_O emissions associated with sugar cane biomass and organic N fertilizers, their indirect N_2_O EF is estimated to be 0.264% (24% × 1.1%).^6^

However, it is worth noting that the direct N_2_O EF measured from sugar cane fields may be lower than the IPCC Tier 1 value of 1%, according to recent field studies conducted in Brazil.^25^ Vasconcelos et al. measured the N_2_O flux under various straw management treatments and N fertilizer types and rates.^26^ They compared their results with those in the literature and found an average N_2_O EF of 0.74 ± 0.6%, based on 15 sugar cane studies conducted in south-central Brazil. In this work, we present results using the direct N_2_O EF of 0.74% as a sensitivity case compared to the baseline case of 1%.

The soil CO_2_ emissions from urea and limestone application are also accounted for by adopting the EFs from GREET 2021, which are calculated based on the carbon contents of these chemicals (i.e., 0.22 g of CO_2_e/g of CaCO_3_ and 0.73 g of CO_2_e/g of urea).^27^

#### 2.1.3 Open-Field Burning of Sugar Cane Straw

Open-field burning of sugar cane straw before manual cutting helps harvest sugar cane, control diseases, and promote growth in the next season. However, open-field burning was identified as a major contributor to WTW GHG and criteria air pollutant emissions.^10^ Brazil will eventually phase out open-field burning in 2030. In fact, some regions of Brazil have already banned this practice, while some producers reported burned areas.

In this study, we assumed 30% as the share of burnt fields in total sugar cane fields, according to the data collected from sugar cane mills. The GREET default EF for open-field burning was employed (Table S3).^6^

#### 2.1.4 Farming Energy Use

The sugar cane mills reported the usage of various fuel blends, such as B10, B20, B30, and B100, during farming. We used that information to calculate the amounts of diesel and biodiesel consumed during sugar cane farming. Sugar cane mills also reported small amounts of gasoline and grid power use during farming (Table S4). Overall, more farming energy is consumed, due to the increasing share of mechanical harvest, compared to the value in GREET 2021 (i.e., 95 000 Btu/metric ton).

#### 2.1.5 Land Use Change Emissions

The expansion of sugar cane farming activity due to increasing ethanol demand may require additional natural land to be converted to farmland, leading to additional GHG emissions due to land use change (LUC). Since the late 2000s, biofuel LCAs have usually accounted for these emissions.

Economic models have been used to simulate the area of land conversion driven by biofuel demand shock scenarios. The estimated land conversion area is then combined with the corresponding carbon EF to calculate the LUC emissions associated with biofuel production. Two databases and models have been developed to estimate the EF of specific land conversions. The first is the Agro-Ecological Zone (AEZ) EF model developed for the California Air Resources Board’s (CARB) Low Carbon Fuel Standard (LCFS), based on data of carbon stocks in different land types.^28^ The second is to utilize sophisticated process-based modeling techniques, such as DAYCENT/CENTURY models,^29,30^ to develop the carbon EF of different land conversions. This approach is exercised partially by the EPA’s Renewable Fuel Standard. The GREET model has an LUC emission module named Carbon Calculator for Land Use Change from Biofuels Production (CCLUB), which is based on the second approach. However, the current version of CCLUB does not have LUC emissions for sugar cane farming.^31^

In this study, we performed a literature review on LUC emissions estimated for Brazilian sugar cane ethanol production. As summarized in Table 1, the LUC GHG emissions ranged from 4.5 to 46 g of CO_2_e/MJ of sugar cane ethanol. This large variation is due to different economic models employed to estimate LUC and different assumptions made about demand shocks and amortization periods by different studies.

**Table 1:** Simulated LUC GHG Emissions for Brazilian Sugar Cane Ethanol Production

study	base year modeled	demand shock (billion gallons of sugar cane ethanol)	economic model used for LUC^c^	emissions (g of CO_2_e/MJ of ethanol)	amortization periods (years)
CARB^32^	2001	2	GTAP	46	30
U.S. EPA^17^	2005	1.6	FAPRI-CARD	4.5	30
CARB^33^	2004	3	GTAP	11.8	30
Valin et al.^34^	2010	1.5	GLOBIOM	17	20
Zhao et al.^35^	2011	1.3^a^	GTAP-BIO	5.5^b^	25

a The demand shock is based on 1.3 billion gallons of sugar-cane-ethanol-derived jet fuel.

b The LUC emissions were estimated for sugar-cane-ethanol-derived jet fuels and back-calculated to obtain the LUC emissions associated with sugar cane ethanol production by using an ethanol-to-jet conversion rate. According to ICAO, 1.633 MJ of ethanol is needed to produce 1 MJ of jet fuel.^36^

c GTAP: Global Trade Analysis Project; FAPRI-CARD: Food and Agricultural Policy Research Institute, the Center for Agricultural and Rural Development; GLOBIOM: Global Biosphere Management Model; GTAP-BIO: GTAP-Biofuels.

### 2.2 Sugar Cane Ethanol Production

#### 2.2.1 Ethanol Production Energy Use

In most cases, the mills are co-located with the farming/growing activities to minimize the sugar cane transportation distance. The energy required for transporting the harvested sugar cane from field to mill is reported as a part of the farming energy use.

In the sugar cane mill, juice is extracted from sugar cane and is either evaporated/concentrated to produce sugar or fermented to produce ethanol. The sugar cane mills have multiple products, including sugar, anhydrous ethanol, hydrous ethanol, and surplus electricity for export (Figure 1). In this analysis, we applied a theoretical conversion approach to define the functional unit to be anhydrous ethanol equivalent by assuming that all of the sugar is converted to ethanol. On the other hand, the RenovaBio calculator (version 7) allocated the upstream GHG emissions among all co-products, based on their energy content (using LHV).^23^

Our use of the theoretical conversion approach is to serve our purpose of conducting LCA on Brazilian sugar cane ethanol and deriving PDFs for key sugar cane ethanol parameters. In reality, sugar cane mills may produce sugar and ethanol, and the owners can determine their product mix based on market demands.

An anhydrous ethanol equivalent value is calculated by adding the anhydrous ethanol yield, 95% of the hydrous ethanol yield (5% water in hydrous ethanol), and 63.3% of the sugar yield (1.58 kg of sugar to produce 1 L of ethanol). It is worth noting that many producers utilize hydrous ethanol as fuel in farm engines (57 of the 59 mills) and ethanol processing facilities (24 of the 59 mills). We assumed that the mills used their own produced ethanol and thus subtracted these uses from the anhydrous equivalent yield to calculate the net yield.

Bagasse is a fibrous residue left behind after sugar cane juice extraction and is combusted at sugar cane mills in biomass boilers to generate steam and electricity to satisfy process needs. Of the 59 plants investigated, 57 combusted their own bagasse for steam and power generation, and 21 purchased additional bagasse from third-party suppliers, with an average transportation distance of 142 km. Small quantities of other biomass purchases were also reported, which include their own straw (3 mills), third-party straw (2 mills), wood chips (6 mills), firewood (14 mills), and forest residues (1 mill). The transportation energy associated with the on site use of their own straw is already reported as a part of the farming energy. Sugar cane mills also reported small amounts of fuel oil and grid power use during the ethanol production stage (Table S5). The combustion of bagasse and straw on-site generates CH_4_ and N_2_O as GHGs, which needs to be accounted for in the WTW emissions (Table S3). The biogenic CO_2_ is excluded from accounting since it is taken up by sugar cane during growth and released back to the atmosphere during combustion.

#### 2.2.2 Co-Product Handling Method

Besides the anhydrous ethanol equivalent as the main product, sugar cane mills generate surplus electricity and bagasse as co-products. In this study, we employed the displacement method as the default co-product handling method.^37^ We assumed that the surplus electricity co-generated from sugar cane mills would displace that from the average Brazilian grid. To deal with the surplus bagasse co-product, we assumed that it is burned in biomass boilers to generate additional electricity to be exported to the grid through the use of the Rankine cycle, as this is becoming a more common practice in sugar cane mills.^18^ Seabra et al. modeled such a system in Aspen HYSYS using a 65 bar/480 °C boiler and calculated the bagasse-to-electricity energy conversion efficiency to be 27.1% (based on the LHV).^18^ Therefore, a surplus bagasse yield of 9.4 kg per metric ton of sugar cane (on a wet basis) translates into 0.25 KWh of additional electricity per gallon of ethanol produced.

Alternatively, Seabra et al. suggested that the avoided emissions due to the bagasse-derived electricity should be calculated based on the EF of fuels in the Brazilian Operating Margin (OM) generation mix, instead of the Brazilian average electricity mix.^18^ They identified natural gas as the predominant fuel in the Brazilian OM generation mix. Therefore, in this study, we present results for a sensitivity case where co-produced electricity would displace that from a natural gas combined cycle (NGCC) power plant.

In addition, we provide results for a sensitivity case where the co-product handling method is energy allocation, in which the upstream GHG emissions associated with sugar cane farming and ethanol production are allocated among sugar cane ethanol, surplus electricity, and surplus bagasse in proportion to their energy contents (based on LHV). This allocation approach could become more appropriate as more electricity is exported from sugar cane mills due to the coutilization of bagasse and straw.^18^

### 2.3 Sugar Cane Ethanol Transportation, Distribution, and End Use

The produced ethanol is transported from sugar cane mills to Brazilian ports via trucks, from Brazilian to U.S. ports via ocean tankers, and from U.S. ports to refueling stations via trucks. The default transportation distances and payloads for various transportation modes were adopted from GREET 2021 (Table S6).

### 2.4 Stochastic Simulation

To estimate the variations associated with sugar cane ethanol CI, we developed PDFs for key input parameters based on the individual plant data from 59 mills and employed the stochastic simulation capacity of the GREET model. We employed the “fitdistrplus” package, a curve-fitting toolbox in R, to identify the best-fit PDF for each key parameter. The toolbox calculates the Akaike information criterion (AIC) score for each of the fitted distributions and ranks the distributions based on this score. We selected the distribution with the lowest AIC score, which indicates the best-fit model.^38^Table 2 presents the best-fit distribution identified for each key parameter, with the mean, 10th percentile (P10), and 90th percentile (P90) values. We quantified the variations in sugar cane ethanol CI by performing Monte Carlo-based stochastic simulations.

**Table 2:** Distribution of Key Parameters

parameter	unit	distribution	mean^a^	P10	P90
farming stage					
total farming energy	mmBtu/metric ton of sugar cane	normal	0.171	0.108	0.225
nitrogen fertilizer	kg/metric ton of sugar cane	normal	1.168	0.482	1.785
CaCO_3_ application	kg/metric ton of sugar cane	normal	10.451	5.020	16.062
vinasse application rate	liters/metric ton of sugar cane	normal	1059.449	530.873	1263.908
filtercake application rate	kg/metric ton of sugar cane	normal	10.056	3.286	15.508
open-field burning	%	exponential	29.8	3.6	78.9
ethanol production stage					
total processing energy	mmBtu/gal of ethanol	normal	0.091	0.048	0.127
electricity surplus	kWh/metric ton of sugar cane	gamma	29.287	∼0	46.003
bagasse surplus	kg/metric ton of sugar cane	gamma	9.381	∼0	27.386
ethanol yield^b^	gal/metric ton of sugar cane	logistic	20.847	18.110	23.520

a The production-weighted average, not the mean value for the derived distribution.

b The net anhydrous ethanol yield equivalent, excluding the use of ethanol on site during sugar cane farming and ethanol processing.

## 3 Results and Discussion

### 3.1 Well-to-Wheels Greenhouse Gas Emissions: Stochastic Results

**Figure 2:**
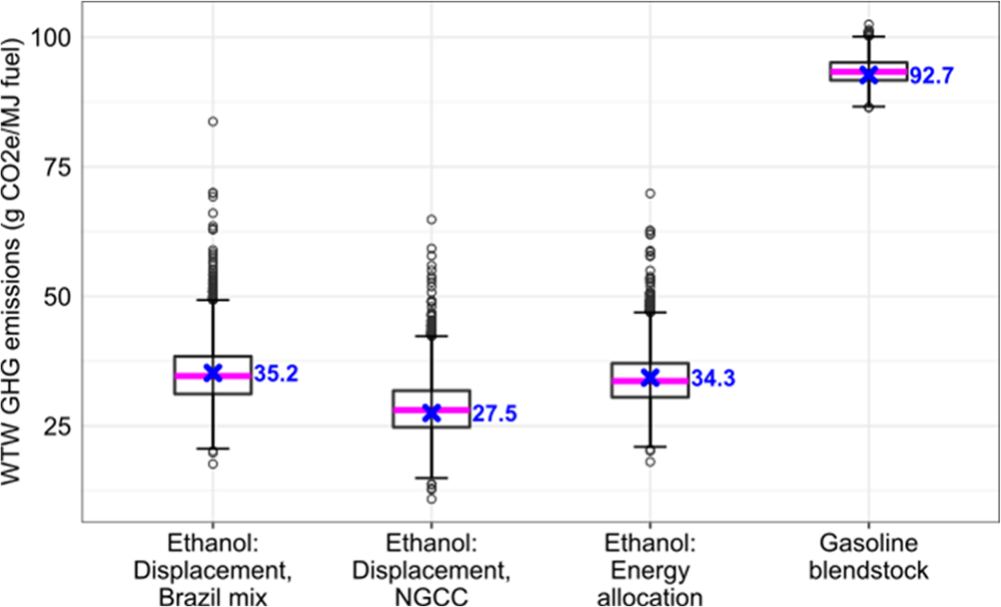
WTW GHG emissions for sugar cane ethanol without LUC, compared to that of gasoline blendstock, from the Monte Carlo stochastic simulations. The lower and upper boundaries of the box represent the 25th percentile (Q1) and the 75th percentile (Q3), respectively. The interquartile range (IQR) is defined as the difference between Q1 and Q3. The pink line indicates the median/50th percentile. The lower and upper error bars represent Q1 – 1.5 × IQR and Q3 + 1.5 × IQR, respectively. The blue cross marks represent the average WTW GHG emissions for each case, calculated using the production-weighted average values, which do not match the median values exactly since many of the derived distributions are not symmetric. The dots represent the results of individual sugar cane mills that have GHG emission values beyond Q1 – 1.5 × IQR or Q3 + 1.5 × IQR.

Figure 2 shows the WTW GHG emissions for sugar cane ethanol under three cases, compared to that of gasoline blendstock. It is noteworthy that the GREET model incorporates predefined PDFs for key petroleum refining parameters, resulting in variations in the WTW GHG emissions of the gasoline blendstock. When stochastic simulations are conducted with GREET, these PDFs are sampled, generating a range of values for gasoline blendstock emissions. However, larger variations are observed in sugar cane ethanol CI, compared to that of gasoline blendstock, from the additional PDFs defined for key sugar cane ethanol parameters (as shown in Table 2).

The average WTW GHG emissions for each case are almost identical to the median GHG emission values generated from Monte Carlo stochastic simulations, indicating that the PDFs identified for key sugar cane ethanol parameters represent the collected data well.

Despite larger variations in results, the CI of sugar cane ethanol is consistently lower compared to that of gasoline blendstock. In the baseline case where the surplus electricity from sugar cane mills displaces that from the average Brazilian grid, sugar cane ethanol achieves a life cycle GHG emissions reduction of 62%, similar to the findings from previous studies.^13,17^ Under the energy allocation case, where 90.6% of the upstream GHG emissions associated with sugar cane farming and ethanol production are allocated to sugar cane ethanol, the GHG reduction potential compared to gasoline blendstock is slightly higher (63%). This is because the displaced Brazilian average electricity mix relies heavily on hydroelectric power, which has a low CI value (30.5 g of CO_2_e/MJ of electricity). Under the case where the surplus electricity from sugar cane mills displaces that from the NGCC power plant, the GHG reduction potential of sugar cane ethanol increases to 70.3% due to the higher CI value of electricity generated by the NGCC power plant (156.3 g of CO_2_e/MJ of delivered electricity).^6,20^ The WTW GHG emissions for this case total 27.5 g of CO_2_e/MJ of ethanol. The difference between this value and the CI value calculated by Seabra et al., 21.3 g of CO_2_e/MJ of ethanol, is due to the assumed end use market; this study assumed that sugar cane ethanol is transported to and used in the U.S., while Seabra et al. assumed that ethanol is transported and used within Brazil.^18^ Subtracting the contribution from ethanol transportation and distribution, the results from the two studies are similar: 20.3 (this study) versus 19.5 g of CO_2_e/MJ of ethanol (Seabra et al.).^18^

### 3.2 Emission Breakdown

**Figure 3:**
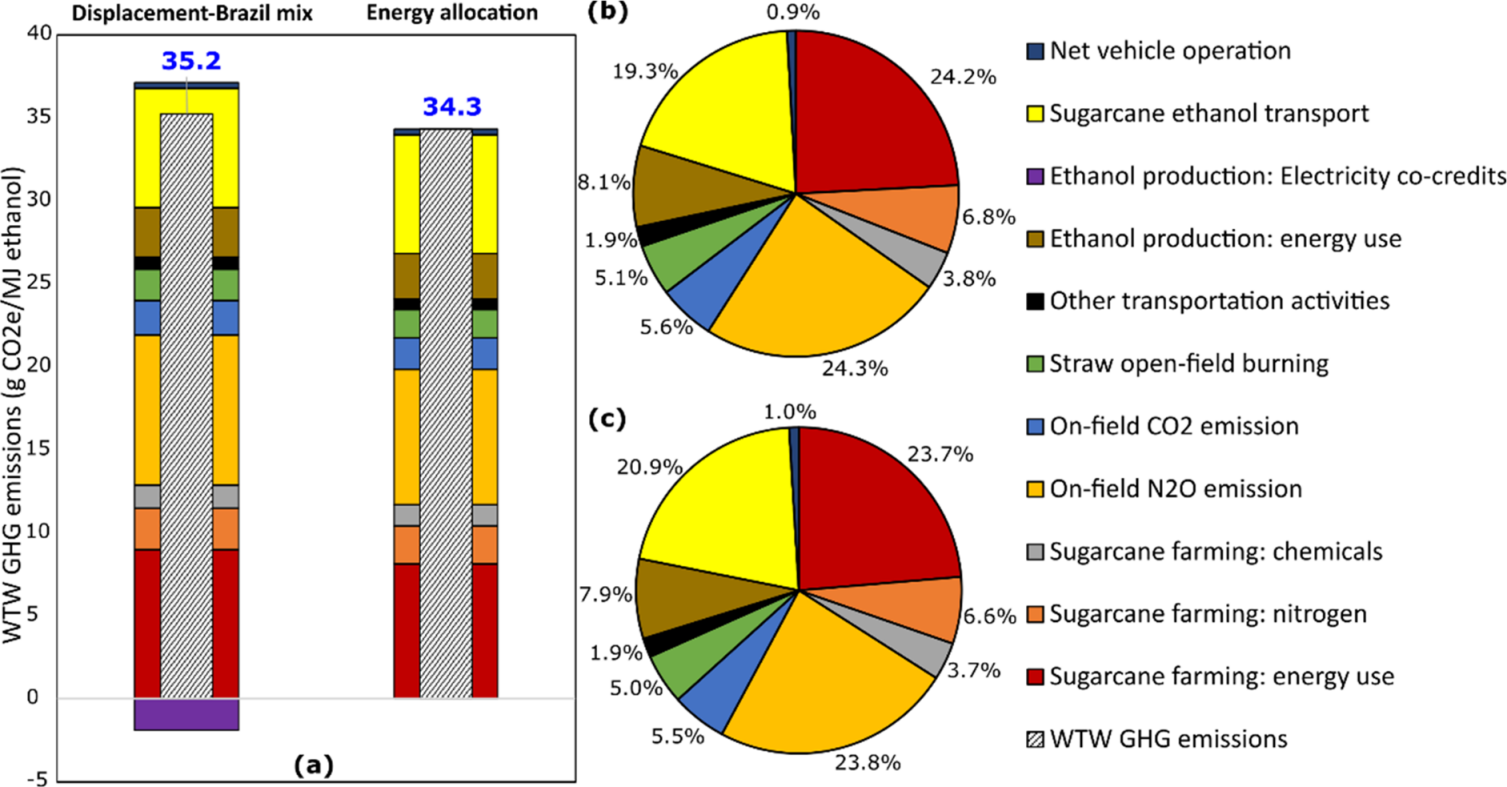
Breakdown of WTW GHG emissions for sugar cane ethanol without LUC (a) by value for each category, (b) by share from each category under the baseline displacement case, and (c) by share from each category under the energy allocation case. In (b), the contribution shares from each category are calculated by excluding the credits generated from displacing the Brazilian average electricity mix (−1.9 g of CO_2_e/MJ).

Figure 3 shows the breakdown of WTW GHG emissions for sugar cane ethanol without LUC. It is worth noting that, for the case in which the co-produced electricity displaces electricity from the NGCC power plant, the breakdown of WTW GHG emissions would be identical to the baseline case, except for the different displaced electricity credits (−1.9 and −9.6 g of CO_2_e/MJ for Brazilian average electricity mix and NGCC electricity, respectively). Therefore, we only show the breakdown for the baseline case here.

The ethanol burned during the vehicle operation stage emits 71.3 g of CO_2_e/MJ, most of which (71.0 g of CO_2_/MJ) is the CO_2_ taken up from the atmosphere during sugar cane growth and thus treated as credits. The net GHG emissions during vehicle operation total only 0.3 g of CO_2_e/MJ, due to the CH_4_ formation from the combustion of ethanol in the engine, contributing merely 0.9% to the WTW GHG emissions, as shown in Figure 3(b).

Under the baseline displacement case, the sugar cane farming stage contributes 69.8% to WTW GHG emissions. The sources of those emissions are as follows:

- On-field N_2_O emissions from N fertilizers and sugar cane biomass residue: 24.3%
- Farming energy use: 24.2%
- Upstream synthetic N fertilizer manufacturing: 6.8%
- On-field CO_2_ emissions from urea and limestone application: 5.6%
- Open-field burning of sugar cane straw: 5.1%
- Upstream manufacturing of other chemicals, including P_2_O_5_ fertilizers, K_2_O fertilizers, limestones, pesticides, and gypsum: 3.8%

To reduce the GHG emissions during sugar cane farming, growers could consider using larger shares of renewable energy to power their agricultural machinery, for example, using diesel fuels with a higher level of biodiesel blending. Growers may also utilize the hydrous ethanol produced at the mills to fuel the agricultural machinery but at the cost of reduced net ethanol yield.

On-field N_2_O is another important source of GHG emissions. As a sensitivity case, when a direct N_2_O EF of 0.74% is employed, compared to the baseline value of 1%, the sugar cane ethanol CI is reduced further by 2.1% to 34.5 g of CO_2_e/MJ. Varying the percentage of sugar cane fields burned also has a significant impact on WTW GHG emissions. If Brazil successfully phases out open-field burning in 2030, it will achieve an additional GHG reduction potential of 5.4% on a WTW basis (33.3 g of CO_2_e/MJ).

The energy consumed during ethanol production contributes only 8.1% to the WTW GHG emissions. This is because the major energy sources for ethanol production are bagasse and straw, the combustion of which generates biogenic CO_2_, which is excluded from GHG accounting. The 8.1% contribution stems from the small amount of fuel oil and grid power consumed during ethanol production (Table S5).

Transportation activities contribute 21.3% to WTW GHG emissions, 19.3% of which is due to sugar cane ethanol transportation and distribution from Brazilian sugar cane mills to U.S. refueling stations. This is mainly because of the long transportation distance from Brazilian ports to U.S. ports via ocean tankers.

The “other transportation activities” in Figure 3(b) together contribute 1.9% to WTW GHG emissions, including the open-channel transportation of vinasse (1.5%), the truck transportation of third-party bagasse to sugar cane mills (0.4%), the truck transportation of filtercake (0%), and the truck transportation of sugar cane to mills (0%). The latter two transportation processes contribute 0% because the energy required for them is reported as a part of the farming energy used by the mills.

In summary, this analysis used the GREET model to calculate the GHG emissions from the farming and ethanol production stages, which are 25.9 and 1.1 g of CO_2_e/MJ of ethanol, respectively. Meanwhile, the RenovaBio calculator produced weighted-average GHG emissions of 23.2 and 1.3 g of CO_2_e/MJ of ethanol among the 59 mills for the farming and ethanol production stages, respectively. Although the two models made different assumptions (as detailed in the Supporting Information), our findings aligned with those generated by the RenovaBio calculator.

For the energy allocation case, no credit is associated with displacing external electricity sources with surplus electricity from the sugar cane mills, since the latter is treated as an energy co-product, receiving GHG emissions allocation from upstream sugar cane farming and ethanol processing. From Figure 3(a), it can be inferred that the contributions of each category to the WTW GHG emissions are similar between the baseline and energy allocation cases. This is because (1) the credit generated from displacing the Brazilian average electricity mix is small for the baseline case and (2) approximately 91% of the upstream GHG emissions associated with sugar cane farming and ethanol production are allocated to sugar cane ethanol under the energy allocation case. This explains why the WTW GHG emissions for these two cases are similar (35.2 versus 34.3 g of CO_2_e/MJ), a result that was also observed by Wang et al.^13^

Due to the great variation in estimated LUC emissions from sugar cane ethanol production, we did not include its contribution in Figures 2 and 3 and associated discussions. However, it is important to understand the impact of such variation on sugar cane ethanol CI. When the estimated LUC GHG emissions are high, e.g., 46 g of CO_2_e/MJ,^32^ the GHG reduction potential of sugar cane ethanol is only 12.4% compared to that of gasoline blendstock. Using regional information for the modeling of the indirect impacts of LUC could improve the accuracy of the estimate but is beyond the scope of this study.^5^ On the other hand, if the more recent and lower LUC GHG value was used (e.g., 5.5 g of CO_2_e/MJ, as in Table 1), sugar cane ethanol would have a WTW GHG reduction of 56.1%.

As many countries and government agencies are rolling out their own biofuel regulatory programs, it is important to track how the CI of biofuels will evolve over time due to the introduction of such programs. Our study utilized data from 67 individual plants submitted to RenovaBio, which went into effect in December 2019; therefore, our results reflect an up-to-date Brazilian sugar cane ethanol industry and establish a reference point to benchmark the future progress in sugar cane ethanol GHG mitigation potential.

## Supporting Information Available

Table S1 for 2020 Brazilian electricity generation mix; Table S2 for N, P_2_O_5_, and K_2_O fertilizer application rates and shares; Table S3 on emission factors of sugar cane straw and bagasse; Tables S4 and S5 on energy usage and share during sugar cane farming and ethanol production, respectively; and Table S6 on default GREET assumptions for sugar cane ethanol transportation (PDF)
Data from 67 individual sugar cane mills submitted to RenovaBio in 2019/2020. (XLSX)
