# Supplementary material for: Life Cycle Greenhouse Gas Emissions of Brazilian Sugar Cane Ethanol Evaluated with the GREET Model Using Data Submitted to RenovaBio
Source: Environ Sci Technol. 2023 Aug 1;57(32):11814–22. doi: 10.1021/acs.est.2c08488 (PMC10433513; doi:10.1021/acs.est.2c08488)
Supplement: Supplementary file 1 — es2c08488_si_001.pdf [file es2c08488_si_001.pdf]

Supplementary information

Life cycle greenhouse gas emissions of Brazilian sugarcane ethanol evaluated with the GREET® model using data submitted to RenovaBio

Xinyu Liu[a], Hoyoung Kwon[a, b], Michael Wang[a], and Don O'Connor[c]

[a] Systems Assessment Center, Energy Systems and Infrastructure Analysis Division, Argonne National Laboratory, 9700 S Cass Ave, Lemont IL, 60439, USA.

[b] Sustainability Sciences Division, Indigo Ag, Inc., 500 Rutherford Avenue, Boston MA, 02129, USA.

[c] S&T Squared Consultants Inc., 11657 Summit Crescent, Delta BC, V4E2Z2, Canada.

This supporting information has 8 pages, containing 6 tables.

Table S1: Electricity generation mix in Brazil in 2020 compared to U.S. mix.

**Fuel**	**U.S. Mix** [1]	**Brazilian Mix** [2]
Residual oil	0.4%	2.9%
Natural gas	39.6%	6.8%
Coal	20.0%	2.1%
Nuclear power	20.4%	2.3%
Biomass	0.3%	9.2%
Hydroelectric	7.4%	65.9%
Others	12.0%	10.9%
T & D loss	4.9%	17.0%

Table S2: Fertilizer use for sugarcane farming.

Nitrogen source	Production-weighted average (kg N/metric ton sugarcane)	Share	Number of mills
Urea	0.7793	66.7%	57
MAP	0.0467	4.0%	47
DAP	0.0001	0.0%	2
Urea-ammonium nitrate solution	0.0049	0.4%	6
Anhydrous ammonia	0.0000	0.0%	0
Ammonium sulphate	0.0491	4.2%	16
Ammonium nitrate	0.2882	24.7%	44
**P_2O_5 source**	**Production-weighted average (kg P_2O_5/metric ton sugarcane)**	**Share**	**Number of mills**
MAP	0.237	46.5%	51
DAP	0.004	0.7%	2
Simple superphosphate	0.268	52.4%	51
Triple superphosphate	0.002	0.4%	4
**K_2O source**	**Production-weighted average (kg K_2O /metric ton sugarcane)**	**Share**	**Number of mills**

**Potash**	1.005	100%	67

Table S3: Emission factors of fuel combustion (unit: g/mmbtu) [1]

**Fuel**	**Straw**	**Straw**	**Bagasse**	**Natural gas**
**Technology**	**Field Burning**	**Small Industrial Boiler**	**Small Industrial Boiler**	**Combined Cycle Powerplant**
**CH_4**	182	9.9	31.7	2.7
**N_2O**	4.7	6.1	4.2	0.2
**CO_2**	111,899	123,124	124,281	115,105
**Biogenic CO_2**	123,619	123,619	124,384	0

Table S4: External farming energy use (all energy use is lower heating value based)

Energy source	Production-weighted average (Btu/metric ton sugarcane)	Share	Number of mills
Diesel	152,952	89.4%	59
Biodiesel	15,763	9.2%	59
Gasoline	81	0.0%	13
Grid power	2,347	1.4%	16

Table S5: Ethanol processing energy (all energy use is lower heating value based)

Energy source	Production-weighted average (Btu/gal ethanol)	Share	Number of mills
Fuel oil	94	0.1%	31
Grid power	169	0.2%	58
Bagasse combusted for steam/power	90,599	99.2%	57
Straw combusted for steam/power	513	0.6%	4

Fifty-seven sugarcane mills used hydrous ethanol as fuel to power their farming operations, with an average usage amount of 3,252 btu/metric ton sugarcane. Compared to other energy sources listed in Table S4, ethanol contributes only 2% to the total energy use

during the farming stage. For the ethanol processing step, only thirty-two mills used hydrous/anhydrous ethanol as process fuel, with a negligible usage amount of 16 btu/gal ethanol. This can be expected since the majority of energy supply during ethanol production stage is from the combustion of bagasse.

Table S6: Key assumptions made by GREET 2021 on transportation and distribution of sugarcane ethanol [1]

From	To	Mode	Payload (short ton)	One-way distance (mile)
Brazilian sugarcane mill	Brazilian port	Truck	25	430
Brazilian port	U.S. port	Ocean tanker	22,000	7,416
U.S. port	U.S. bulk terminal	Truck	25	100
U.S. bulk terminal	U.S. refueling station	Truck	25	30

The four transportation stages in Table S6, namely, ethanol transportation within Brazil, from Brazilian ports to U.S. ports, from U.S ports to U.S. bulk terminals, and from U.S. bulk terminals to U.S. refueling stations, contribute 30%, 61%, 7%, and 2%, respectively, to the overall GHG emissions associated with sugarcane ethanol transportation and distribution.

Differences in Assumptions between GREET and RenovaBio Calculator

The key methodological difference is that RenovaBio utilized energy allocation between co-products, including hydrous ethanol, anhydrous ethanol, and sugar. On the other hand, our analysis adopted a theoretical conversion approach by calculating an anhydrous ethanol equivalent yield. This is because one of the purposes of this analysis is to derive probability density functions (PDFs) for key parameters based on individual mill data. Since sugarcane mills can produce various co-products and the owners can determine their product mix based on many factors, including market demands and production capabilities, deriving PDFs for each individual product in the product mix would not make much sense. Due to this methodological difference, it is very difficult to compare the results from this study to those calculated with RenovaBio on an apple-to-apple basis. However, we summarized the key assumption differences employed by the two models:

1) RenovaBio utilizes the Ecoinvent 3.1 database (2014) with the location specified to "Rest of world" to calculate the upstream GHG emissions associated with farming inputs/chemicals manufacturing; while in this analysis, we configured GREET model to accommodate some Brazilian specific parameters, e.g., Brazilian electricity mix.
2) Both models used the empirical approach to calculate N_2O emission factors (EFs) from the field, but the EFs employed are different. For RenovaBio calculator, the N_2O EF for synthetic fertilizer is 1.325%; while the GREET model employed the most up to date N_2O EFs of 1.374%, based on IPCC 2019 refinement.

3) The RenovaBio calculator uses 1.12 kg nitrogen/tonne sugarcane for the nitrogen content of the crop residues; while in GREET model, the nitrogen content of the crop residues is calculated based on the sugarcane straw yield and straw harvest rate to be 1.19 kg nitrogen/tonne of sugarcane.
4) For organic soil supplements, such as vinasse and filtercake, the N_2O EF employed by the RenovaBio calculator is based on that of organic fertilizers to be 1.425%; while the N_2O EF employed by the GREET model is based on that of biomass residue to be 1.264%.
5) The combustion EFs for various fuel types employed by RenovaBio calculator and GREET are different.

References

(1) Wang, M.; Elgowainy, A.; Lee, U.; Bafana, A.; Banerjee, S.; Benavides, P. T.; Bobba, P.; Burnham, A.; Cai, H.; Gracida-Alvarez, U. R.; et al. *Summary of Expansions and Updates in GREET® 2021*; Argonne, IL (United States), 2021.

(2) EIA. U.S. Energy Information Administration International Brazil https://www.eia.gov/international/data/country/BRA (accessed Jun 14, 2022).
